# Continued use of low-dose aspirin does not increase the risk of bleeding during or after endoscopic submucosal dissection for early gastric cancer

**DOI:** 10.1007/s10120-013-0305-3

**Published:** 2013-10-19

**Authors:** Yoji Sanomura, Shiro Oka, Shinji Tanaka, Norifumi Numata, Makoto Higashiyama, Hiroyuki Kanao, Shigeto Yoshida, Yoshitaka Ueno, Kazuaki Chayama

**Affiliations:** 1 Department of Endoscopy, Hiroshima University Hospital, 1-2-3 Kasumi, Minami-ku, Hiroshima, 734-8551 Japan; 2 Department of Gastroenterology and Metabolism, Hiroshima University Hospital, 1-2-3 Kasumi, Minami-ku, Hiroshima, 734-8551 Japan

**Keywords:** Early gastric cancer, ESD, Aspirin, Complication, Bleeding

## Abstract

**Background:**

Although recent guidelines for endoscopic submucosal dissection (ESD) as treatment for early gastric cancer (EGC) recommend noninterruption of low-dose aspirin (LDA) perioperatively, this strategy is controversial. It was our practice to interrupt LDA therapy 5–7 days before to ESD until December 2010, when we instituted the new guidelines and performed ESD without interrupting LDA therapy. Our purpose in this study was to confirm the validity of noninterrupted use of LDA in patients undergoing ESD for EGC.

**Methods:**

We studied 78 consecutive patients with 94 EGCs who were routinely taking LDA and were treated by ESD at Hiroshima University Hospital between April 2005 and June 2012. The patients were of two groups: those in whom LDA was interrupted perioperatively (53 patients with 66 EGCs) and those in whom LDA was continued perioperatively (25 patients with 28 EGCs).

**Results:**

The complete resection rate was 92.4 % (61/66) in the LDA-interrupted group and 100 % (28/28) in the LDA-continued group. Incidences of poor bleeding control during the procedure and bleeding after procedure were 10.6 % (7/66) and 4.8 % (3/66), respectively, in the LDA-interrupted group and 7.1 % (2/28) and 3.6 % (1/28) in the LDA-continued group. Two patients in the interrupted-LDA group suffered cerebrovascular infarction before ESD, and 2 patients in this group suffered acute myocardial infarction after ESD.

**Conclusions:**

Our data suggest that continued use of LDA does not increase the risk of bleeding during or after ESD for EGC and does decrease the risk of ischemic events.

## Introduction

Early gastric cancer (EGC) is defined as tumor invasion confined to the mucosa or submucosa, regardless of the presence of lymph node metastasis [1]. Endoscopic submucosal dissection (ESD), which allows en bloc resection of EGC, is commonly performed as treatment in Japan [2–6]. The 2010 Japanese Gastric Cancer Association (JGCA) guidelines for treatment expanded the condition of curative resection for ESD to include submucosal invasion <500 μm (SM1) [7]. We have reported the clinical validity of ESD without additional surgical resection for SM1-GCs of the differentiated type that are <30 mm in diameter and without vessel involvement [8, 9] as well as the usefulness of ESD for EGC with ulceration [10–12].

Although the safety of the procedure has been substantiated, complications such as bleeding and perforation remain problematic. Bleeding after gastric ESD is reported in as many as 5 % of patients and can occur several days after the procedure, even after discharge from the hospital [2, 13–17]. We have reported patients at high risk for post-ESD bleeding in gastric epithelial neoplasm to be those undergoing dialysis, those in whom operation time is >75 min, and those in whom bleeding during ESD is poorly controlled [14].

Recently, despite a plateau in the total number of patients in Japan, the incidence of gastric cancer has increased, owing to the expanding lifespan of the general population [18]. Accordingly, the number of patients taking antiplatelet medicines including low-dose aspirin (LDA) has increased as a result of the increase in the number of patients with ischemic heart disease, cerebrovascular disease, and arteriosclerosis obliterans. The American Society for Gastrointestinal Endoscopy (ASGE), European Society of Gastrointestinal Endoscopy (ESGE), and British Society for Gastroenterology have all published guidelines for the management of anticoagulant and antiplatelet therapies in patients undergoing endoscopic procedures [19–21]. In Japan as well, endoscopic guidelines for the management of anticoagulant and antiplatelet therapies were published in 2012 and include performance of gastroenterological endoscopy without interruption of LDA therapy in patients who use LDA only at high risk for thromboembolic events [22]. However, there are insufficient data supporting this strategy. Also, the issue of bleeding risk after gastric ESD remains controversial for patients routinely using LDA; only a few studies have been conducted [23–27]. In 2010, Hiroshima University Hospital defined that patients using LDA for ischemic heart disease should not interrupt it perioperatively. Therefore, since December 2010 we have performed gastric ESD without interrupting all the anti-thrombogenic agents. Because we have treated consecutive patients under both the traditional guideline (interrupted use of LDA) and the present guideline (continued use of LDA) as defined by Hiroshima University Hospital, we were able to obtain comparative data and thus conducted a retrospective study of patients who underwent ESD for EGC to confirm the validity of continued use of LDA.

## Methods

### Patients

Subjects identified for the study were 78 consecutive patients on LDA therapy and treated by ESD for 94 EGCs at Hiroshima University Hospital between April 2005 and June 2012. Patients were from a total group of 1,183 patients with 1,432 gastric epithelial neoplasms treated by ESD during the same period. Traditionally, we have interrupted therapy 5–7 days before gastric ESD for all patients using LDA; however, since December 2010, we have followed the Hiroshima University Hospital guidelines and have thus performed gastric ESD under continued use of antiplatelet agents including LDA in all cases. Patients taking warfarin for the prevention of thromboembolic disease were switched to heparin starting 4 days before ESD in principle. The 78 patients comprised two groups: an LDA-interrupted group (53 patients with 66 EGCs treated between April 2005 and November 2010) and an LDA-continued group (25 patients with 28 EGCs treated between December 2010 and June 2012). Use of patient data for the purpose of this study was approved by the Institutional Review Board of Hiroshima University.

### Patient characteristics

Clinical characteristics of the 78 patients with EGC resected by ESD are shown in total and per group in Table 1. LDA was used for ischemic heart disease in 49.1 and 60.0 % of patients in the LDA-interrupted group and LDA-continued group, respectively; cerebrovascular disease in 32.1 and 32.0 %; and arteriosclerosis obliterans in 9.4 and 4.0 % of patients, respectively. Comorbidities were common: hypertension and diabetes mellitus were often present, with liver cirrhosis and/or chronic renal failure requiring hemodialysis present in only a few patients. There was no significant difference between the two groups in age, sex ratio, indications for LDA, comorbidities, or routine use of anticoagulant/antiplatelet agents other than LDA. Patients taking warfarin were taking it in combination with LDA; warfarin was replaced by heparin in all such cases. The main antiplatelet agents used in combination with LDA were ticlopidine, clopidogrel, and cilostazol. There was no between-group difference in the use of anticoagulants or antiplatelet agents.

**Table 1:** Clinical characteristics of study patients with early gastric cancer resected by endoscopic submucosal dissection (ESD) in total and per group

Characteristic	Total patients *n* = 78 (April 2005–June 2012)	LDA-interrupted group *n* = 53 (April 2005–November 2010)	LDA-continued group *n* = 25 (December 2010–June 2012)	*P* value
Age, mean (SD), years	73.7 (8.9)	72.7 (9.1)	75.9 (8.2)	n.s.
Sex, male (%)	64 (82.1)	44 (83.0)	20 (80.0)	n.s.
Reasons for use of LDA (%)				n.s.
Ischemic heart disease	41 (52.6)	26 (49.1)	15 (60.0)	
Cerebrovascular disease	25 (32.1)	17 (32.1)	8 (32.0)	
ASO	6 (7.7)	5 (9.4)	1 (4.0)	
Other	6 (7.7)	5 (9.4)	1 (4.0)	
Comorbidities (%)				
Hypertension				n.s
Present	63 (80.8)	44 (83.0)	19 (76.0)	
Absent	15 (19.2)	9 (17.0)	6 (24.0)	
Diabetes mellitus				n.s
Present	18 (23.1)	14 (26.4)	4 (16.0)	
Absent	60 (76.9)	39 (73.6)	21 (84.0)	
Liver cirrhosis				n.s
Present	2 (2.6)	1 (1.9)	1 (4.0)	
Absent	76 (97.4)	52 (98.1)	24 (96.0)	
Dialysis necessary				n.s
Yes	3 (3.8)	3 (5.7)	0 (0.0)	
No	75 (96.2)	50 (94.3)	25 (100.0)	
Use of other anticoagulants and/or antiplatelets (%)				n.s
Yes	25 (32.1)	17 (32.1)	8 (32.0)	
Anticoagulants: warfarin	6 (7.7)	6 (11.3)	0 (0.0)	
Antiplatelets: ticlopidine	5 (6.4)	4 (7.5)	1 (4.0)	
Clopidogrel	7 (9.0)	1 (1.9)	7 (28.0)	
Cilostazol	5 (6.4)	5 (9.4)	0 (0.0)	
Beraprost	1 (1.3)	1 (1.9)	0 (0.0)	
Ethyl icosapentate	1 (1.3)	1 (1.9)	0 (0.0)	
No	53 (67.9)	36 (67.9)	17 (68.0)	

*ESD* endoscopic submucosal dissection, *LDA* low-dose aspirin, *SD* standard deviation, *ASO* arteriosclerosis obliterans

### Lesion characteristics

Characteristics of the EGCs resected by ESD are shown in total and per group in Table 2. Tumors were 16.6 and 18.3 mm in size in the LDA-interrupted group and LDA-continued group, respectively, with maximum ulcer diameter of 38.5 and 46.0 mm, respectively. There was no between-group difference in tumor location, macroscopic type, tumor size, maximum ulcer diameter, histology, depth of invasion, or presence of ulceration.

**Table 2:** Characteristics of early gastric cancers resected by ESD in total and per study group

Characteristic	Total lesions *n* = 94 (April 2005–June 2012)	LDA-interrupted group *n* = 66 (April 2005–November 2010)	LDA-continued group *n* = 28 (December 2010–June 2012)	*P* value
Tumor location (%)				n.s.
Upper	19 (20.2)	11 (16.7)	8 (28.6)	
Middle	24 (25.5)	20 (30.3)	4 (14.3)	
Lower	51 (54.3)	35 (53.0)	16 (57.1)	
Macroscopic type (%)				n.s
Depressed	43 (45.7)	29 (43.9)	14 (50.0)	
Nondepressed	51 (54.3)	37 (56.1)	14 (50.0)	
Tumor size (mm) (SD)	17.1 (10.9)	16.6 (9.9)	18.3 (13.2)	n.s
Maximum diameter of ulcer (mm) (SD)	40.7 (22.9)	38.5 (16.0)	46.0 (34.0)	n.s
Histology (%)				n.s
Differentiated	90 (93.8)	63 (95.5)	27 (96.4)	
Undifferentiated	6 (6.2)	3 (4.5)	1 (3.6)	
Depth of invasion (%)				n.s
Mucosa	83 (86.5)	57 (86.4)	26 (92.9)	
Submucosa	11 (13.5)	9 (13.6)	2 (7.1)	
Ulceration (%)				n.s
Present	9 (9.6)	6 (9.1)	3 (10.7)	
Absent	85 (90.4)	60 (90.9)	25 (89.3)	

*ESD* endoscopic submucosal dissection, *LDA* low-dose aspirin, *SD* standard deviation

### Indications and ESD procedure

Endoscopic submucosal dissection was principally indicated for apparently node-negative EGC as follows: differentiated-type intramucosal adenocarcinoma without ulceration regardless of size, differentiated-type intramucosal adenocarcinoma with ulceration but ≤3 cm in size, or undifferentiated-type intramucosal adenocarcinoma without ulceration and ≤2 cm in size.

Endoscopic submucosal dissection was performed with the use of a single-channel endoscope (GIF-H260, GIF-H260Z, or GIF-Q260J; Olympus; or EG-450RD5; Fujifilm Medical) or a two-channel endoscope (GIF-2TQ260M; Olympus; or EG-450D5; Fujifilm Medical) by four endoscopists. Several spots were marked by argon plasma coagulation 5 to 10 mm outside the margin of the cancer lesion. After injection of 10 % glycerin solution and 5 % fructose with 0.0025 % epinephrine into the submucosa, an initial incision was made with a needle knife outside the line of spots. We used mainly an IT knife, IT knife2, or Hook knife (Olympus), which was then inserted into the initial incision, and electrosurgical current was applied with the use of an electrosurgical generator (ICC 200, VIO 300D, ERBE, or ESG-100; Olympus) to complete the circumferential mucosal incision around the lesion, as previously reported [2–6, 8–18, 26–32]. An IT knife or IT knife2 was used to exfoliate the submucosa with coagulation current. Injection was repeated as needed, and further resection was carried out to ensure total removal of the lesion.

At the end of the ESD procedure, all exposed vessels on the artificial ulcer were coagulated with the use of hemostatic forceps (FD-410LR, Olympus; or HDB2418W-W, Pentax). Beginning on the day of ESD, rabeprazole (20 mg/day), sodium alginate (120 ml/day), and aluminum hydroxide (40 ml/day) were administered. We consistently undertook second-look endoscopy on the day after ESD, and we coagulated all exposed vessels on the artificial ulcer regardless of whether bleeding was present [14]. After hemostasis was confirmed, the patient was permitted a light meal in the evening. Written informed consent was obtained from all patients for ESD.

### Histopathological examination and curability after ESD

Histopathological examination was based on the 2010 Japanese Classification of Gastric Carcinoma issued by the JGCA [1]. The entire resected specimen was cut into parallel 2-mm-thick sections and examined under hematoxylin and eosin staining for detailed analysis, including analysis of the deepest invasive portion containing infiltrating cancer cells. GCs are classified as differentiated or undifferentiated. The former type includes well-differentiated tubular adenocarcinoma, moderately differentiated tubular adenocarcinoma, and papillary adenocarcinoma; the latter includes poorly differentiated adenocarcinoma, signet-ring cell carcinoma, and mucinous adenocarcinoma. En bloc resection was defined as resection in a single piece. Complete resection was defined as en bloc resection of a tumor that was shown to be free of cancer cells at both the horizontal and vertical cut ends.

The resection was judged as curative when all the following criteria were met: en bloc removal, tumor size ≤2 cm, differentiated type, pT1a, negative horizontal margin (HM0), negative vertical margin (VM0), and no lymphovascular infiltration (ly(−), v(−)). Curative resection for EGCs that fall under the expanded indications was defined as follows: en bloc resection, HM0, VM0, ly(−), v(−) as well as (a) tumor size ≤2 cm, differentiated type, pT1a, and ulceration (UL)(−); (b) tumor size ≤3 cm, differentiated type, pT1a, UL(−); (c) tumor size ≤2 cm, undifferentiated type, pT1a, UL(−); or (d) tumor size ≤3 cm, differentiated type, pT1b (SM1, ≤0.5 mm from the muscularis mucosae) [7].

### Evaluation of outcomes

For each group, we investigated the rates of en bloc resection, complete resection, curability, bleeding control during the procedure, bleeding after the procedure, perforation, operation time, and ischemic events before and after procedure, and we compared rates between the two groups.

Good control of bleeding during ESD was defined as no visible bleeding during the procedure or trivial bleeding that stopped spontaneously or was easily controlled by a few sessions of coagulation. Poor control of bleeding during ESD was defined as bleeding that required multiple coagulation sessions (≥10 sessions) [14]. Bleeding after ESD was defined as bleeding manifested by a fall in the hemoglobin level of 2 g/dl or more below the most recent preoperative level, observation of any bleeding source, or massive melena [33].

### Statistical analysis

Quantitative data are expressed as mean and standard deviation (SD) or percentages. Differences in values were analyzed by χ^2^ test with Yates correction or by Student’s *t* test. *P* < 0.05 was considered statistically significant.

## Results

Outcomes of ESD for EGC in patients who use LDA are shown in Table 3. En bloc resection and complete resection rates were 95.5 % (63/66) and 92.4 % (61/66), respectively, in the LDA-interrupted group and 100 % (28/28) and 100 % (28/28) in the LDA-continued group. The curative resection rate and expanded curative resection rate were 68.2 % (45/66) and 15.2 % (10/66), respectively, in LDA-interrupted group, and 78.6 % (22/28) and 14.3 % (4/28) in the LDA-continued group. Poor bleeding control during the procedure and bleeding after the procedure occurred in 10.6 % (7/66) and 4.8 % (3/66) of patients, respectively, in the LDA-interrupted group and 7.1 % (2/28) and 3.6 % (1/28) of patients in the LDA-continued group. The perforation rate was 4.8 % (3/66) in the LDA-interrupted group and 0 % (0/28) in the LDA-continued group. Operation time was 45.3 min in the LDA-interrupted group and 49.6 min in the LDA-continued group. There were no between-group differences in the en bloc resection rate, complete resection rate, curability, poor bleeding control during procedure, bleeding after the procedure, perforation rate, or operation time.

**Table 3:** Outcomes of ESD for early gastric cancers in total and per study group

Characteristic	Total lesions *n* = 94 (April 2005–June 2012)	LDA-interrupted group *n* = 66 (April 2005–November 2010)	LDA-continued group *n* = 28 (December 2010–June 2012)	*P* value
En bloc resection (%)				n.s.
Yes	91 (96.8)	63 (95.5)	28 (100.0)	
No	3 (3.2)	3 (4.5)	0 (0.0)	
Complete resection (%)				n.s
Yes	89 (94.7)	61 (92.4)	28 (100.0)	
No	5 (5.3)	5 (7.6)	0 (0.0)	
Curability (%)				n.s
Curative resection	67 (71.3)	45 (68.2)	22 (78.6)	
Expanded curative resection	14 (14.9)	10 (15.2)	4 (14.3)	
Noncurative resection	13 (13.8)	11 (16.7)	2 (7.1)	
Bleeding control during procedure (%)				n.s
Good	85 (90.4)	59 (89.4)	26 (92.9)	
Poor	9 (9.6)	7 (10.6)	2 (7.1)	
Bleeding after procedure (%)				n.s
Yes	4 (4.3)	3 (4.8)	1 (3.6)	
No	90 (95.7)	63 (95.2)	27 (96.4)	
Perforation (%)				n.s
Yes	3 (3.2)	3 (4.8)	0 (0.0)	
No	91 (96.8)	63 (95.2)	28 (100.0)	
Operation time (min) (SD)	46.4 (53.3)	45.3 (51.3)	49.6 (58.8)	n.s

*ESD* endoscopic submucosal dissection, *LDA* low dose aspirin, *SD* standard deviation

Bleeding after the procedure occurred after an average of 8 days in three patients in the LDA-interrupted group and after 8 h in one patient in the LDA-continued group; in all cases, we coagulated all exposed bleeding vessels on the artificial ulcer, and no rebleeding was observed.

### Ischemic events before and after the procedure

Two patients in the LDA-interrupted group suffered cerebrovascular infarction before the procedure, one 2 days and the other 4 days before the scheduled ESD; thus, ESD was not performed. Two patients in this same group suffered an acute myocardial infarction after ESD. One of the two patients had suffered a myocardial infarction 10 years earlier, and the second acute myocardial infarction occurred 5 h after the ESD procedure. We performed coronary angiography 6 h after the procedure and observed 100 % stenosis of the left main trunk. We placed a stent at the site of stenosis after aspiration of the thrombus. Intraaortic balloon pumping and percutaneous cardiopulmonary support were initiated to restore the patient’s continuously falling blood pressure, but the patient died 18 days after ESD. No ischemic events occurred in the LDA-continued group during the perioperative period.

## Discussion

We set out to gather comparative data from our large university hospital that would substantiate the recommendation for continuance of LDA therapy immediately before, during, and after ESD. Our study showed no significant difference in the perioperative bleeding rate between patients in whom LDA therapy was suspended perioperatively and patients in whom LDA therapy was not suspended. However, there was a difference in the occurrence of ischemic events. Four patients in whom LDA therapy was suspended perioperatively suffered an ischemic event, two before ESD and two after ESD. Our findings support perioperative continuance of LDA therapy in patients undergoing ESD.

Results of other studies concerning the risk of bleeding in patients taking LDA and undergoing endoscopic resection have varied. Case–control studies that in total have included 29,606 patients undergoing colonoscopic polypectomy showed no increased risk of hemorrhage with use of LDA [20, 34–38]. As late as 2009, however, Fujishiro et al. [23] reported interruption of antiplatelet therapy in most patients 1 week before gastric ESD. It has often been reported in cases in which the anti-thrombogenic agent was interrupted 1 week before to ESD that the incidence of accidental bleeding did not increase [24, 25, 31, 32]; however, it has also been reported that the risk of post-procedure bleeding increased despite the interruption [26].

The guidelines of the various clinical associations are not consistent regarding the types of cases in which LDA should be interrupted for gastric ESD. According to the ASGE guidelines, LDA should be continued for treatment under gastroenterological endoscopy even when the risk of bleeding is high [19]. The ESGE guidelines match the ASGE LDA continuation guidelines in principle but recommend a 5-day interruption for endoscopic resection and ESD when the risk of thromboembolism is low. The new guidelines published in Japan in July 2012 state that it is appropriate to perform high-risk (bleeding risk) gastroenterological endoscopy without interrupting LDA in patients who take LDA as a single agent because of a high risk of thromboembolism; however, a sufficient study has not yet been conducted. Recently, Cho et al. [27] reported that LDA should be interrupted for patients at low risk for thromboembolism development because the risk of bleeding after gastric ESD increases significantly in patients in whom LDA is not interrupted, although Lim et al. [25] reported no difference in the post-gastric ESD bleeding rate between patients in whom anti-thrombogenic agents were continued and those in whom they were interrupted. It is important to note that because all these reports are of retrospective studies, the results should be interpreted under the understanding that they could be subject to selection bias. This restriction could be true of our results as well. However, the different perioperative approaches to LDA therapy in our patients were based on time periods, not on patient selection or clinical preference. Thus, our results are reliable. It is not possible for any group to avoid the possibility of bias altogether because a randomized study of interruption versus noninterruption of LDA would not be clinically ethical because of the risk of ischemic events.

Ticlopidine and clopidogrel are used worldwide to decrease the risk of ischemic events in various patients, with clopidogrel reported to be useful in combination with LDA after placement of a drug-eluting stent [39]. Regarding the use of combined therapy, Cho et al. [27] reported an increased risk of post-ESD bleeding when LDA was used in combination with clopidogrel rather than used alone, whereas Lim et al. [25] reported no change in the risk of post-ESD bleeding when other antiplatelet agents were used in combination with LDA. We studied results of cases in which warfarin, ticlopidine, or clopidogrel was used in combination with LDA and found no increase in the rate of post-ESD bleeding. Few reports exist regarding whether bleeding rates after gastric ESD increase when other anti-thrombogenic or anticoagulant agents are used in combination with LDA; data from a large number of cases are needed.

For cases in which LDA is used, not only is the matter of post-ESD bleeding of concern but also the matter of bleeding during the procedure. We previously reported that poor bleeding control during ESD became a risk factor for post-ESD bleeding [14]. The present study is the first to consider bleeding during a procedure with respect to noninterruption of LDA therapy. When we analyzed bleeding control during the procedure, we found the incidence of poor bleeding control during procedure to be somewhat greater in the LDA-interrupted group than in the LDA-continued group, and bleeding control essentially the same in the two groups. In terms of outcomes of gastric ESD, the en bloc resection rate and complete resection rate were quite high in both groups, substantiating noninterruption of LDA.

With aging of the population, we can expect to encounter an increased number of EGC patients with ischemic disease. Among our study patients were two who had suffered cerebral infarction before ESD and two who suffered acute cardiac infarction after ESD, both in the LDA-interrupted group. This finding indicates a potential increase in the occurrence of ischemic events when LDA is interrupted.

Our data suggest that continued use of LDA does not increase the risk of bleeding during or after ESD for EGC and may decrease the risk of ischemic events. The limitation of this study is the retrospective analysis from a single center and a small number of patients. Our results are clinically important, and a much larger multi-center study should be conducted to further evaluate the management of anti-thrombogenic agents in relationship to ESD of EGC.
